# The Repetition of the Dose

**Published:** 1882-12

**Authors:** C. Lippe

**Affiliations:** New York


### THE REPETITION OF THE DOSE.

C. Lippe, M. D., New York.

*Read before the Central New York Homœopathic Society.*

Paragraphs 246, 247, and 248, with the notes, appear to be contradictory to the practice of many of the present followers of Hahnemann whose experience has taught them the almost miraculous results from the single dose.

It must be remembered that Hahnemann's usual potency was the 30th, and from his experience with that potency he found the necessity of the repetitions he speaks of.

In sec. 245 the law is laid down: "Both in acute and chronic diseases every perceptible amelioration that takes place, making continual progress, though of ever so feeble a nature, is a state which as long as it endures formally forbids the repetition of any medicine whatever, because the one already taken by the patient has not yet produced all the good that may result from it. Every fresh dose of a remedy, even of the one last administered and which had till then proved salutary, would have no effect but that of disturbing the operation of the cure." Here, then, is the law of repetition very clearly expressed; yet the next paragraph, 246, apparently contradicts the former. But it must be read carefully, and I call attention to this extract from the paragraph: * * * "First, when the remedy has been chosen with due circumspection—that is, strikingly homœopathic; secondly, when it is administered in the highest development, the least revolting to the vital power, and yet sufficiently energetic to influence it; and thirdly, when such a subtile, energetic dose of the best selected remedy *is repeated at the most suitable intervals* which experience has determined for accelerating the cure, yet, in fulfilling this condition, it is requisite that the vital power to be influenced to the production of the similar medicinal disease may not be excited to disagreeable counteraction." Here are three conditions necessary before a repetition of the dose, not, however, laid down as a *law*, but subject to the experience of the practitioner. It is worthy of remark that on reading the *Organon,* when a law is to be promulgated there is no appeal to experience or mode of practice, but such a law is clearly and concisely stated as in sec. 245. In sec. 246 will be found one of the greatest arguments for the single dose. Notice the last words of the paragraph. * * * "May not be excited to disagreeable counteraction." Did the homœopathic remedy act curatively by its *direct action*, then the oftener repeated the sooner would the disease be subdued; but these remedies are curative only by the power of *re-action;* and a repetition of the dose, as a rule, would not allow of this reaction, for the vital forces would be under the constant irritation of the repeated doses, interrupting the progressive curative action already begun.

The late Dr. Carroll Dunham in his *Science of Therapeutics* says: "We suppose Hahnemann meant as follows: 'If amelioration follows a dose of medicine, do not repeat the dose until the amelioration ceases to progress; *then*, if the symptoms be the same as before, though mitigated in severity, repeat the dose. If the symptoms be different, study the case anew and make another selection of the remedy.'".

It is often a study when to repeat a dose or when to change the remedy. If the amelioration is steady, if reaction has set in, it would be the height of folly to interfere. But suppose there appears to be no amelioration, new symptoms are added to those already existing, what then? Your pathological knowledge now will tell you if these symptoms are an indication of the advance of the disease; your materia medica if the remedy is showing its reaction powerfully. In the first case, your selection was bad, and a fresh study of the case is to be made; in the second instance, wait for the full reaction.

It is the universal acknowledgment of those who have followed implicitly Hahnemann's *laws* that their success has been great, but failure was a sure accompaniment to a divergence from the narrow path of truth. Dr. Dunham, on the same page quoted above, gives testimony to the same effect.

With the higher potencies, experience has taught the reaction is more lasting than with the lower. Such as have not made the test are not competent judges. If in an acute case a wise selection has been made, an improvement is seen after the administration of the highest potency in a very short time in the most grave cases, slight, to be sure, and generally first in the mental condition. Now rest quietly, give no medicine as long as the improvement lasts; if that ceases and no new symptoms arise, give another dose of a higher potency; if new symptoms arise, find out if they show an unfavorable progress of the disease or if they belong to the remedy. In the first case a new selection must be made; in the second nothing is needed. In chronic cases the same rule holds good, always supposing the true homœopathic remedy has been selected, and many of these cases absolutely get well with a single dose.

There is another point to which attention is particularly called. Experience and experiment have taught that if we desire to obtain a full and correct knowledge of the power of drugs to produce their specific effects, they must be administered to the healthy in the single dose, for the fact is, that the most reliable provings have been made in this manner. If the dose should be repeated before the full action and reaction of the drug has been accomplished—before the gradual return to the normal condition which has been disturbed by the proving, which resembles the return to health after an illness—the action of the drug is disturbed and the proving will not be as valuable or accurate. Just in the same proportion if the dose administered is repeated before the full reaction is obtained, just in the same proportion is the cure retarded, with the addition of new complications which may arise from a needless repetition.

